# Application of Artificial Intelligence Techniques to Predict Risk of Recurrence of Breast Cancer: A Systematic Review

**DOI:** 10.3390/jpm12091496

**Published:** 2022-09-13

**Authors:** Claudia Mazo, Claudia Aura, Arman Rahman, William M. Gallagher, Catherine Mooney

**Affiliations:** 1 UCD School of Computer Science, University College Dublin, D04 V1W8 Dublin, Ireland; 2 UCD School of Biomolecular and Biomedical Science, UCD Conway Institute, University College Dublin, D04 V1W8 Dublin, Ireland

**Keywords:** breast cancer, risk of recurrence, artificial intelligence, machine learning, feature predictors, systematic review

## Abstract

Breast cancer is the most common disease among women, with over 2.1 million new diagnoses each year worldwide. About 30% of patients initially presenting with early stage disease have a recurrence of cancer within 10 years. Predicting who will have a recurrence and who will not remains challenging, with consequent implications for associated treatment. Artificial intelligence strategies that can predict the risk of recurrence of breast cancer could help breast cancer clinicians avoid ineffective overtreatment. Despite its significance, most breast cancer recurrence datasets are insufficiently large, not publicly available, or imbalanced, making these studies more difficult. This systematic review investigates the role of artificial intelligence in the prediction of breast cancer recurrence. We summarise common techniques, features, training and testing methodologies, metrics, and discuss current challenges relating to implementation in clinical practice. We systematically reviewed works published between 1 January 2011 and 1 November 2021 using the methodology of Kitchenham and Charter. We leveraged Springer, Google Scholar, PubMed, and IEEE search engines. This review found three areas that require further work. First, there is no agreement on artificial intelligence methodologies, feature predictors, or assessment metrics. Second, issues such as sampling strategies, missing data, and class imbalance problems are rarely addressed or discussed. Third, representative datasets for breast cancer recurrence are scarce, which hinders model validation and deployment. We conclude that predicting breast cancer recurrence remains an open problem despite the use of artificial intelligence.

## 1. Introduction

Cancer mortality rates are falling due to recent advancements around earlier diagnosis and improved therapeutic options. However, further work is still needed considering that breast cancer is still one of the most frequent cancers in Europe, and it is the second leading cause of cancer mortality [1,2]. A breast cancer diagnosis has an impact on an individual’s health, lifestyle, job, and family life [3]. It carries not only the danger of severe morbidity and mortality, but also the risk of physical and psychosocial consequences that persist after therapy is completed. Economic hardship owing to lost working hours and healthcare costs might be an additional burden induced by this disease [4]. As a result, breast cancer clinicians require precise tools to aid in clinical decision-making in order to enhance patient prognosis, survival, and quality of life while lowering associated costs [4].

Women who have had early stage breast cancer are at risk of recurrence, either locally, regionally or at distant sites. Approximately 30% of patients develop cancer again within 10 years—although 80% of these occur within five years of diagnosis [5]. At the moment, it is difficult to discern between those who will have a recurrence and those who will not.

Artificial intelligence techniques are emerging to resolve medical issues such as diagnosis, prognosis, drug design, and testing [6,7,8,9,10,11] in different specialties. Specifically in breast cancer, artificial intelligence techniques have been used for the diagnosis [12] and prognosis [13] of breast cancer, the classification and quantification of immunohistochemistry stained images [14,15,16], and the prediction of pathological complete response (pCR) to neoadjuvant chemotherapy [17,18], offering the opportunity for personalised care, improved therapy response rates, reduced adverse effects, and decreased costs of unnecessary treatment.

Researchers would like to find papers of interest, contributions, and evidence in order to prevent repetition and to enhance their results given the variety of approaches, investigations, and published papers on predicting the risk of breast cancer recurrence using artificial intelligence. There are some reviews on the risk of recurrence in breast cancer based on identifying machine learning techniques and comparing results [19]. However, artificial intelligence techniques are advancing so fast that it is necessary to update these reviews frequently. This review aims to provide an overview of the prediction of breast cancer recurrence using artificial intelligence techniques. It adds to the existing literature by summarising artificial intelligence techniques used, the most appropriate features, common training and testing methodologies, the common evaluation metrics, and system implementation in clinical practice.

## 2. Method

The study of the application of artificial intelligence techniques to predict the risk of recurrence of breast cancer was conducted according to the methodology of Kitchenham and Charter [20]. Kitchenham adapted the medical guidelines for systematic literature review to software engineering [21] and the guidelines of Kitchenham and Charter have been adapted to reflect the specific issues associated with software engineering research. This methodology composed of three stages: (i) planning the review—related works and need for the review, and research question; (ii) conducting the review—data sources, and extracting data and synthesis, and (iii) results—what artificial intelligence techniques are being used, what type of features are being used, what are the common training and testing methodologies, what model evaluation metrics are being used, and what systems have been implemented in clinical practice, or validated in a real-world context.

### 2.1. Planning the Review

#### 2.1.1. Related Works and Needs for the Review

This review aims to explore the literature surrounding artificial intelligence techniques, features, training and testing methodologies, model evaluation metrics, and use of artificial intelligence to predict the risk of recurrence of breast cancer. Considering there are different strategies, studies, and a significant amount of published papers on predicting the risk of recurrence of breast cancer using artificial intelligence, researchers need to identify publications of interest, contributions, and evidence in order to avoid repetition and to improve their results. In recognition of the gap within the existing literature, we conducted a systematic literature review using electronic bibliographic databases from January 2011 to November 2021.

#### 2.1.2. Research Questions

The research questions that we aimed to address were:

- RQ1: What artificial intelligence techniques have been used to predict the risk of recurrence of breast cancer and what is their performance?
- RQ2: What type of features have been used?
- RQ3: What were the common training and testing methodologies used?
- RQ4: What model evaluation metrics have been used, and what are the advantages and disadvantages of these metrics?
- RQ5: What systems have been implemented in clinical practice, or validated in a real-world context?

### 2.2. Conducting the Review

#### 2.2.1. Data Sources

We conducted a systematic search of the literature in the following scientific and academic databases and search engines: Springer, Google Scholar, PubMed, and IEEE. The searches were conducted in English. Only studies using artificial intelligence techniques to predict the risk of recurrence of breast cancer were selected.

Selecting appropriate search terms was a key step; keywords that were too broad yielded an unwieldy number of irrelevant publications, but terms that were too particular seemed to overlook significant research. This required some experimentation with a range of terms to select the key words for a broad and inclusive review of the application of artificial intelligence techniques to predict the risk of recurrence of breast cancer. We performed a search using the following query:

*("Predicting Breast Cancer Recurrence" OR "Risk of Recurrence Breast Cancer Prediction" OR "Recurrence Prediction Breast Cancer") AND ("Artificial Intelligence" OR "Machine Learning")*

**Figure 1:**
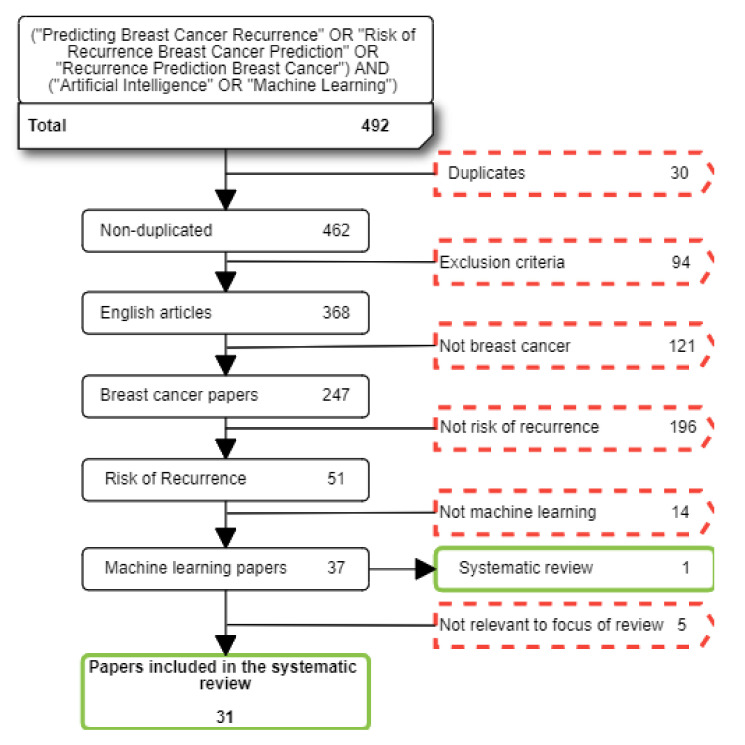
Flow diagram summarising the literature search, inclusion, and exclusion process. Red dotted squares correspond to excluded paper; green continuous squares correspond to selected papers.

We searched for studies reported between 1st January 2011 and 1st November 2021. A total of 492 papers were found at this stage before excluding irrelevant papers. Table 1 shows the exclusion and inclusion criteria which were applied to papers based on the purpose of our systematic review. After applying these exclusion criteria an additional five papers which focused on the impact of breast cancer diagnostics on the risk of recurrence were considered to be beyond the scope of this review. A total of 31 papers were finally selected in this stage (see Figure 1).

#### 2.2.2. Extracting Data and Synthesis

In order to verify the quality of the selected studies, each study that met the inclusion criteria was abstracted by a reviewer and a questionnaire was completed for each paper. Each question was designed to elicit information about potential limitations in the quality of the study. The evaluation questions were: (i) Was the artificial intelligence solution well described (what, how, who, where)?; (ii) Was the study population (i.e., number of patients, availability, target population, and years of recurrence) well described?; (iii) Was the data type (i.e., patient, clinical, molecular, or medical images) well described?; (iv) Were the evaluation metrics well described? Answers that showed quality problems were assessed to see whether they were significant enough to diminish confidence in the results.

## 3. Results

### 3.1. RQ1: What Artificial Intelligence Techniques Have Been Used to Predict the Risk of Recurrence of Breast Cancer and What Is Their Performance?

Artificial intelligence has made a substantial contribution to cancer research. Despite the fact that deep learning classifiers have dominated many research areas, traditional machine learning models are more widely used (n=26; 83.9%) than deep learning (n=5; 16.1%) in the field of breast cancer recurrence risk prediction, according to our review. This could be related to the difficulty of getting large datasets and conducting retrospective analyses over a long period of time to train models. Most of studies compared a number of methods and then selected the best one (n=22; 71.0%); three studies, 9.7%, proposed an ensemble method among the evaluated methods; and a small number tried only a single method (n=6; 19.4%), see Table 2.

**Figure 2:**
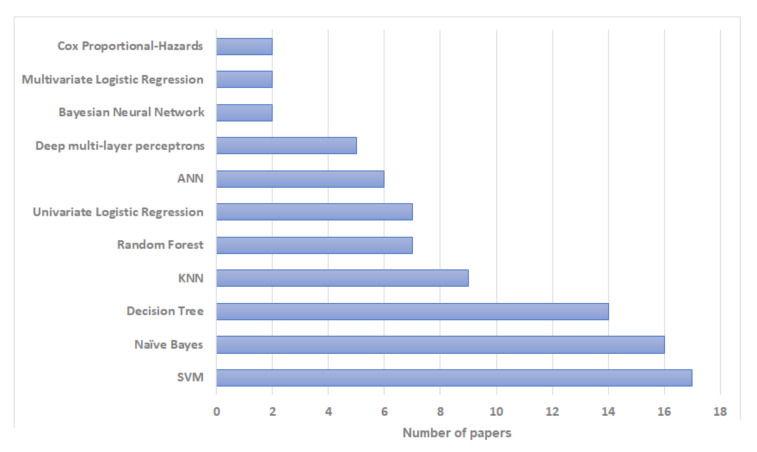
Bar plot showing the frequency of use of different artificial intelligence algorithms in papers included in our review.

**Figure 3:**
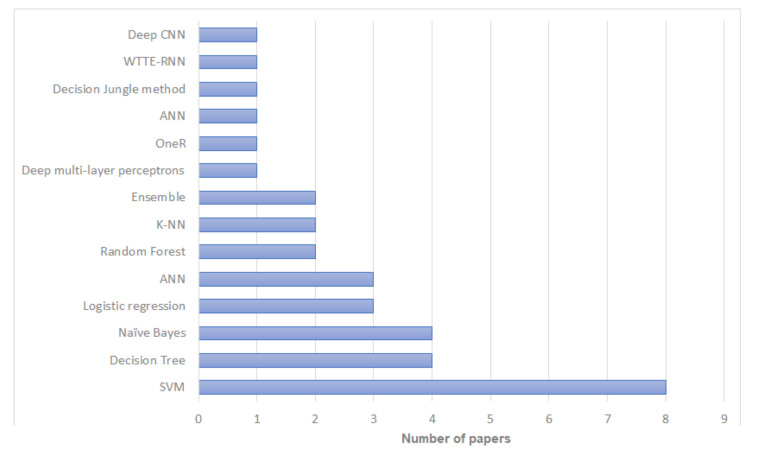
Artificial intelligence algorithms with the highest prediction accuracy in papers included in our review.

According to our review, among machine learning methods, Support Vector Machines (SVM) have been used most commonly for breast cancer recurrence risk prediction—used in 17 studies. Naïve Bayes and Decision Trees have also been used extensively in this research, with 16 and 14 studies respectively. Bayesian Neural Networks and Multivariate Logistic Regression were the least used with only two studies for each. The distribution of literature based on artificial intelligence prediction methods is shown in Figure 2 and Table 2. In terms of the reliability and the prediction outcomes, SVM had the best accuracy in most cases (n=8; 25.8%) followed by Decision Trees and Naïve Bayes (n=4; 12.9%). The distribution of literature based on algorithms with the highest prediction accuracy is shown in Figure 3 and Table 2. However, the prediction outcomes are based on each study independently and they are not directly comparable due to the use of different datasets and/or evaluation metrics.

**Table 2:** Table showing the use of artificial intelligence algorithms in papers included in our review.

Publication	Algorithms	Training Set	Validation Set	Best Algorithm	Best Algorithm
		(Total/Recurrence)	(Total/Recurrence)		Accuracy
Lg et al. [22]	Decision Tree C4.5, SVM, ANN	547/117	10-fold Cross-Validation (CV)	SVM	Accuracy: 0.957, Sensitivity: 0.971, Specificity: 0.945
Pritom et al. [23]	Naïve Bayes, Decision Tree C4.5, and SVM	198/47	10-fold CV	SVM	75.75% accuracy
Aline et al. [24]	Deep multi-layer perceptrons	152/—	168/—	Deep multi-layer perceptrons	AUC: 0.63 low, 0.59 intermediate, and 0.75 high risk
Mosayebi et al. [25]	Deep Multilayer Perceptron ANN, Bayesian Neural Network, LVQ neural network, KPCA-SVM, Random Forest, and Decision Tree C5.0	7874/5471	nested 5-fold CV	Decision Tree C5.0	Accuracy: 0.819, Sensitivity: 0.869, and Specificity: 0.777
Alzubi et al. [26]	Decision Tree J48, Naïve Bayes, bagging, logistic regression, SVM, KNN, MLP, PART, and OneR	142/—	10-fold cross- validation	OneR	Accuracy: 0.1408, Sensitivity: 0.901, and Specificity: 0.72
Witteveen et al. [27]	Logistic regression and Bayesian Networks	72,638/37,230	24,063/12,308	Logistic regression	C-statistic: 0.71
Cirkovic et al. [28]	Naive Bayes, Decision tree C4.5, SVM polynomial kernel, logistic regression, K-NN, and ANN	146/—	live-oneout CV	ANN	AUC: 0.847
Ramkumar et al. [29]	SVM with linear and Radial kernel, Basis function kernel, Random Forest, Elastic Net, Multilayer perceptron, Normal mixture modeling	298/—	196/—	SVM Radial Kernel	AUC: 0.678
Almuhaidib et al. [30]	Random Forest, Decision tree, and Naïve Bayes	194/46	10-fold CV	Random Forest	Accuracy 0.6522, Sensitivity 0.6250, and Specificity 0.659
Rosa Mendoza et al. [31]	Univariate and multivariate logistic regression	215/—	—/—	Multivariate logistic regression	Sensitivity: 0.74 and Specificity 0.97
Wang et al. [32]	Random Forest, SVM with linear kernel, logistic regression, Stochastic Gradient Descent Classifier (SGDC), Naïve Bayes, KNN	4513/312	1934/134	KNN	AUC: 0.888
Chou et al. [33]	ANN, Decision trees, Logistic regression, Composite models of DT-ANN and DT-LR	370/—	387/—	ANN	Accuracy: 70.93
Li et al. [34]	Linear regression	84/—	—/—	Linear regression	AUC: 0.88
Kim et al. [35]	Random Forest, Decision Jungle, NN, Naïve Bayes, and SVM	301/—	76/—	Decision Jungle	Accuracy: 0.90
Kim et al. [36]	Weibull Time To Event Recurrent Neural Network (WTTE- RNN)	10,494/—	2623/—	WTTE- RNN	Accuracy: 0.90
Chakradeo et al. [37]	Multiple Linear Regression, SVM (RBF kernel), and Decision Tree	198/46	CV	SVM	Accuracy: 0.97, Precision: 0.93, and Recall: 0.91
Rana et al. [38]	SVM, Logistic Regression, KNN, and Naive Bayes	194/46	CV	KNN	Accuracy: 0.72
Mohebian et al. [39]	Bagged Decision Tree (BDT), SVM, Decision Tree, Multilayer perceptron neural network	579/112	4-fold CV	Ensemble Learning	AUC: 0.90
Eun et al. [40]	Random Forest, Decision Tree, KNN, Linear discriminant analysis (LDA), linear SVM, and Naïve Bayes	130/21	5-fold CV	Random Forest	AUC: 0.94
Bhargava et al. [41]	Decision Tree J48	286/85	10-fold cross validation	Decision Tree J48	Precision: 0.76
Adeyemi et al. [42]	Naïve Bayes, Decision trees C4.5, and SVM the stack ensemble models, Base (B) and Meta (M). B: Naïve Bayes, SVM and M: C4.5; B: Naïve Bayes, SVM and M: C4.5; B: SVM, C4.5 and M: Naïve Bayes	201/85	10-fold CV	Ensemble method: B: Naïve Bayes, SVM and M: C4.5	Precision Recurrence: 0.554 and No-Recurrence: 0.765
Yang et al. [43]	AdaBoost and Cost sensitive learning	1061/37	3-fold CV	Ensemble method	ROC: 0.907
Massafra et al. [44]	Naïve Bayesian, Random Forest, and SVM	256/—	10-fold CV	SVM	Accuracy: 80.39
Turkki et al. [45]	Deep CNN	868/—	431/—	Deep CNN	C-index: 0.60
Kabiraj et al. [46]	Naïve Bayes	275/85	10-fold CV	Naïve Bayes	Accuracy: 73.81
Sakri et al. [47]	Naïve Bayes, Decisio Tree, and KNN	198/47	10-fold CV	Naïve Bayes	Precision Recurrence: 0.814 and No-Recurrence: 0.381
Lou et al. [48]	Multi-layer perceptron neural network ANN, KNN, SVM, and Naïve Bayesian	798/—	171/—	ANN	AUC: 0.998
Ojha and Goel [49]	clustering algorithms: K-means, EM, PAM, and Fuzzy c-means classification algorithms: SVM, Decision Tree C5.0, Naïve bayes, and KNN	194/46	10 fold cross validation	SVM and Decision Tree C5.0	Accuracy: 0.81
Kim et al. [50]	SVM, ANN, and Cox-proportional hazard regression model	679/45	204	SVM	AUC: 0.85
Woojae et al. [51]	Naïve Bayesian	475/31	204	Naïve Bayesian	AUC: 0.81
Zain et al. [52]	Naïve Bayes, KNN, and Fast Decision Tree (REPTree)	198/47	10 fold cross validation	Naïve Bayes	F-Score: 0.721

### 3.2. RQ2: What Type of Features Have Been Used?

**Figure 4:**
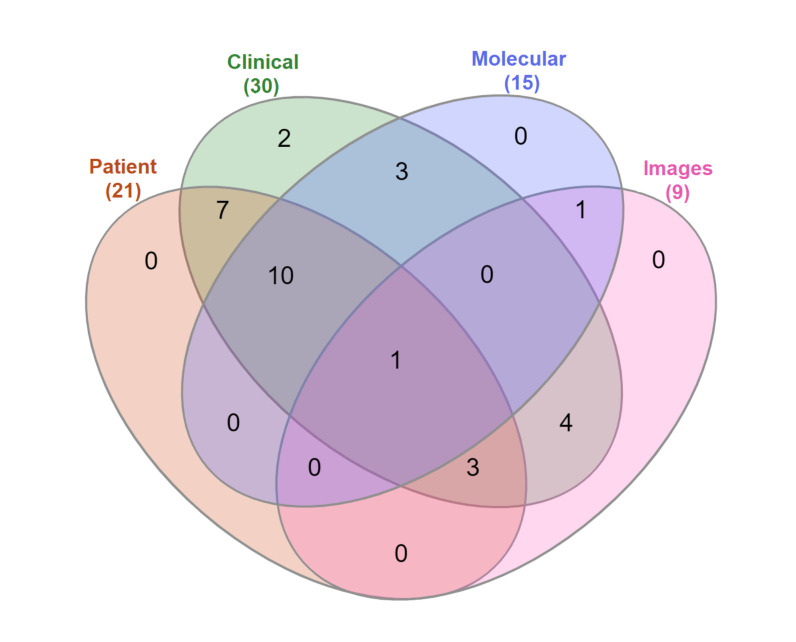
Venn diagram showing the different data types used in research included in this review.

The type of data used to train a prediction model can significantly affect the performance of the model, and impact on the model’s reliability and prediction outcomes [53]. Most of the research studies reviewed in this work included clinical data (n=30; 96.8%), followed by patient demographic information (n=21; 67.7%), molecular data (n=15; 48.4%), and pathological image data (n=9; 29.0%). Most research combined multiple types of data, as shown in Figure 4, which illustrates the distribution of papers based on the type of data used to train the prediction model.

Regarding patient characteristics, the majority of studies (n=17; 54.8%) identified age at diagnosis as an important predictor, followed by menopausal status (n=7; 22.6%) and family history of breast cancer (n=4; 12.9%). The distribution of patient characteristics is summarised in Table 3.

Regarding clinical and molecular features, we used the classification proposed in the Eighth Edition of the AJCC Cancer Staging Manual [54,55]. The distribution of clinical and molecular characteristics is summarised in Table 4.

Concerning anatomic staging, the majority of studies (n=29; 93.5%) identified nodal status as an important predictor of recurrence, followed by tumour size (n=28; 90.3%) and MRI scan diagnostic features (n=12; 38.7%). The distribution of anatomic staging is summarised in Table 4. The results confirm that the pathologic staging via the TNM (T describes the size of the tumour and any spread of cancer into nearby tissue; N describes spread of cancer to nearby lymph nodes; and M describes metastasis) system is a highly discriminant feature in terms of breast cancer prediction and risk of recurrence. Concerning prognostic stage characteristics, tumour grade was identified as an important predictor according to most of the studies (n=21; 67.7%); followed by hormone receptor status (n=15; 48.4%), and tumour invasion (n=13; 41.9%). The distribution of prognostic stage characteristics is summarised in Table 4. This ranking is coherent with the interrelationships between tumour grade, hormone receptor status, and tumour invasion and their connection with pathologic TNM staging in breast cancer [56]

Regarding medical image features, the majority of studies (n=12; 38.7%) employed Magnetic Resonance Imaging (MRI) as an important input source, followed by histopathological images from Fine Needle Aspirate (FNA) (n=6; 19.4%), and images from Tissue Microarray (TMA) samples (n=1; 3.2%). The distribution of images characteristics is summarised in Table 5. Studies using MRI are based on texture features [34,40]—mean pixel intensity, standard deviation, mean proportion of positive pixels, entropy, skewness, and kurtosis. Studies using images from FNA samples utilised the same public dataset, the Wisconsin Prognostic Breast Cancer dataset from the UCI machine learning repository, and the same cell evaluation set of features [37,52]—radius, texture, perimeter, area, smoothness, compactness, concavity, concave points, symmetry, and fractal dimension. Turkki et al. [45] leveraged images from a TMA comprised of primary tumour tissue and utilised a feature extractor with a CNN—different features present in an image such as edges, vertical lines, horizontal lines, bends, texture, colour, and among others.

### 3.3. RQ3: What Were the Common Training and Testing Methodologies Used?

We assessed dataset size, degree of class balance, validation strategies, sample techniques, and data handling strategy, all of which have a direct influence on training and testing performance [57]. Furthermore, we determined whether they had a public or private dataset that is beneficial for reproducibility, Explainable Artificial Intelligence (XAI) [58]. A summary is presented in Table 6.

#### 3.3.1. Dataset Size and Class Balance

With the introduction of computer systems, the digitisation of clinical examination and medical records in healthcare systems has become a standard and widely accepted practice. However, there are challenges with breast cancer recurrence dataset size and class balance, given that around 30% of patients develop a recurrence of breast cancer within 10 years and the difficulty in keeping records of follow-up patients for a long period (e.g., changes in the patient’s domicile and centre of treatment, failure to attend follow-up appointments, patient death). This challenge is revealed in this review study. The majority of works relies on dataset sizes ranging from 100–500 cases (*n* = 17; 51.61%) [23,24,26,28,30,31,37,38,40,41,42,44,46,47,49,52] and only four research papers reference a dataset with >2000 incident cases [25,27,32,36]. Furthermore, none of the studies had balanced data (see Table 6) as the ground-truth is typically unbalanced, with the recurring class being less than 30%. Special strategies are required in artificial intelligence to manage restricted and unbalanced data to lessen the impact on training and testing procedures (e.g., data augmentation techniques); however, there is no indication of their application in the research that we reviewed.

#### 3.3.2. Sampling Strategies

Data selection is an important phase in artificial intelligence training and testing procedures. Selecting data has a direct impact on the performance of the resulting model [53]. Sampling is a strategy for picking instances/patients/registers in order to make statistical inferences from them or in our case, to train and test artificial intelligence models. Probability sampling is a sampling technique in which researchers choose samples from a larger population using a method based on probability theory. In our analysis, four different types of probability sampling techniques were observed (see Table 6): (i) simple random sampling which is entirely by chance (n=20; 64.5%); (ii) stratified random sampling, in which the population is divided into subgroups that share a common characteristic (n=8; 25.8%); (iii) cluster random sampling, in which the population is divided into subgroups known as clusters that are randomly selected to be included in the study (n=1; 3.2%); and (iv) systematic random sampling, which uses regular intervals (n=2; 6.5%). The use of a sampling technique improves the degree of representativeness and generalisation power of the artificial intelligence models generated [59]. However, it may be time consuming and tedious.

#### 3.3.3. Data Handling Strategies

Health data often contain a lot of missing values. Missing values can be caused by failure to record data due to a lack of standards or by data corruption. Handling missing data is crucial during data preprocessing since many artificial intelligence algorithms do not handle missing values, thereby affecting their performance. In our analysis, excluding cases with incomplete data was the most commonly used strategy (n=24; 77.4%). This strategy contributes to training a robust model by removing any missing values. However, there is a significant loss of information, and the strategy performs badly if the percentage of missing values is high in contrast to the whole dataset. There are two particular cases in which strategies to impute missing values were used (n=2; 6.5%): continuous variable substitution using Expectation Maximization [22] and predictive value imputation [44]. Medical data are particularly sensitive, and such strategies might result in data leakage or outliers. In four studies, no evidence of a data handling strategy was found, and there were not details regarding the management of cases with missing values [23,24,27,28], which complicates replication and further comparison of results by other researchers. A summary is presented in Table 6.

#### 3.3.4. Validation Strategies

A cross-validation approach was employed by the majority of studies (n=18; 58.1%). Cross-validation is an internal validation strategy that is common with small datasets since it involves splitting one input dataset into parts/holds—with some parts used for training the classifier (training data), and the remainder used for validation (test data). This approach is repeated until each part has been used as testing data at least once. However, cross-validation cannot ensure the quality of a machine learning model since possibly biased or imbalanced data leads to a biased evaluation. External model validation was used in 11 research papers (35.5%), which test the original prediction model on a set of independently derived external datasets, to validate the performance of a model that was trained on initial input data. There are only two studies (6.5%) which do not describe any validation strategy, which complicates the replication and further comparison of results by other researchers (see Table 6).

#### 3.3.5. Dataset Availability

Many artificial intelligence solutions are trained and tested on private/restricted datasets, such as those holding sensitive patient information [60] or belong to private companies that cannot or do not want to make their data publicly available. Dataset availability is essential for repeatability, transparency, and to verifying one’s own implementation of the other approaches, as well as explaining differing results [58,61]. Governments, as well as health and research institutes, participate in Open Science by hosting publicly available datasets that may be used further. However, dataset availability remains a concern in breast cancer recurrence cohorts, as evidenced by this review in which less than half of the studies (n=13; 41.9%) used public datasets and the remainder used private datasets (see Table 6).

### 3.4. RQ4: What Model Evaluation Metrics Have Been Used, and What Are the Advantages and Disadvantages of These Metrics?

Metrics used to evaluate prediction models are key tools used to select one model or other. Choosing the wrong metric for model assessment will result in an incorrect model selection or, in the worst case, being deceived about the predicted model performance. Choosing an appropriate metric is challenging in artificial intelligence in general, but is particularly difficult for imbalanced classification/prediction problems [62]. In contrast to traditional evaluation metrics, which evaluate all classes equally, unbalanced classification/prediction issues often regard classification mistakes with the minority class as more important than those with the majority class [62]. As a result, performance measures focusing on the minority class may be necessary, which is difficult because it corresponds to the minority class where we often lack the observations required to train an effective model. The objective is to avoid or reduce bias towards the performance on cases poorly represented due to the available data sample.

According to our review, the top six evaluation metrics used for breast cancer recurrence risk prediction are: (i) specificity (n=20; 64.5%); (ii) sensitivity (n=19; 61.3%); (iii) accuracy (n=18; 58.1%); (iv) AUC (n=16; 51.6%); (v) F-Score (n=8; 25.8%); and (vi) precision (n=7; 22.6%). The distribution of the evaluation metrics used for breast cancer recurrence risk prediction is summarised in Table 7. Sensitivity, specificity, precision, and F-Score may be useful for imbalanced classification/prediction because they are based on the confusion matrix that provides more insight into not only the performance of a predictive model, but also which classes are being predicted correctly, which incorrectly, and what types of errors are being made [62]. However, reporting classification/prediction accuracy for a severely imbalanced classification problem could be dangerously misleading. A ROC curve is a diagnostic plot that calculates the false positive rate and true positive rate for a series of predictions made by the model at different thresholds to summarize the behaviour of the model [62]. AUC is useful for imbalanced classification/prediction issues, specifically for problems where both classes are important.

### 3.5. RQ5: What Systems Have Been Implemented in Clinical Practice, or Validated in a Real-World Context?

In this systematic review, there is no evidence that any of the studies have been implemented in clinical practice, or validated in a real-world context, with all of them being described as theoretical solutions. Indeed, despite the popularity of artificial intelligence solutions, we are concerned that there are several barriers preventing the integration of these novel methods into clinical practice.

Artificial intelligence techniques to predict the risk of recurrence of breast cancer could potentially improve the following areas: healthcare system services, decision-making time, and health-related quality of life for patients, as well as lower healthcare expenses and medical mistake rates [63]. However, similar to other healthcare innovations, artificial intelligence solutions should be rigorously assessed. Consequently, certain controlled trials are required before being implemented in clinical practice. Medical mistakes are both costly and hazardous, an error in predicting the risk of recurrence of breast cancer might have catastrophic implications for health-related quality of life and outcome [64]. This may explain, in part, the lack of artificial intelligence solutions to predict the risk of recurrence of breast cancer available.

## 4. Discussion

In this study, we systematically reviewed the literature published between 1st January 2011 and 1st November 2021 on the application of artificial intelligence techniques to predict the risk of recurrence of breast cancer. We considered papers that were written in English. Our study shows dataset availability, training and validation description—dataset size, balanced data, sampling strategy, data handling strategy—artificial intelligence methods used, the best algorithm performance, features used—patient, clinical, molecular, and pathological information—and evaluation metrics.

H&E image-based risk prediction using deep learning and machine learning has potential clinical value if used as a pre-test for selecting patients for expensive gene-based molecular assays. Molecular tests are not available in many low to medium income countries and, where they are available, the tests are expensive and conducted centrally so there is generally a long turnover time. Couture et al. [65] has compared image-based classifiers with the PAM50 molecular test (PAM50 is a 50-gene signature that classifies breast cancer into five molecular intrinsic subtypes for risk prediction). They used deep learning algorithms on breast cancer H&E images to classify tumour grade, ER status, PAM50 intrinsic subtype, histologic subtype, and risk of recurrence score (ROR-PT). It is important to mention that the attributes that these deep learning approaches detect such as receptor status, intrinsic subtype or even risk of recurrence, to predict complex image properties are not visually apparent to pathologists from H&E images. Besides a high degree of concordance between molecular test and image analysis in relation to predict of ER positivity: these authors showed that PAM50 RNA-based molecular subtype (Basal-like vs. non-Basal-like), and risk of recurrence score (ROR-PT) could be predicted using deep learning approaches with approximately 75–80% accuracy, with ductal vs. lobular histologic subtype accuracy as high as 94%. A similar approach using both deep learning and machine learning algorithms was also employed by Whitney et al. [66] to analyse routine H&E-stained images of early-stage ER+ breast cancer patients to predict corresponding Oncotype DX recurrence risk. Oncotype DX is a 21 gene assay that is currently employed to assess the risk of early-stage ER positive (+) breast cancers, and guide clinicians in the decision of whether or not to use chemotherapy. Using the deep learning extracted features of nuclear morphology in the stroma and epithelium followed by four different supervised machine learning classifiers—the authors have clearly stratified patients into low, intermediate, and high-risk groups of recurrence as conducted by the OncotypeDx. Their classifier models trained on low vs. high and the low with intermediate vs. high ODx cases generated the highest classification accuracy (79% and 85%) on the validation set. These studies demonstrate that AI-based techniques have a bright future in the clinic as a tool in combination with molecular assays. These algorithms can create an inexpensive, rapid predictor of low and high-risk categories for early stage breast cancer based on H&E images alone. However, it is evident that this is still an open problem after performing this review. This conclusion is based on the following issues related to our research questions found during the review process.

In answer to our first research question to identify and critically appraise what artificial intelligence techniques are being used to predict the risk of recurrence of breast cancer and their targeted outcomes, there is clear evidence of the effectiveness of artificial intelligence in healthcare to improve patient diagnosis, prevention, and treatment, as well as cost efficiency and equality in health services, transforming the practice of medicine [67,68,69]. This type of evidence is also needed for artificial intelligence systems that forecast the likelihood of recurrence in breast cancer patients. Our systematic review returned 31 papers on artificial intelligence techniques which support the risk of recurrence of breast cancer. One of our findings was that Machine Learning techniques excluding Deep Learning methods are more widely used than Deep Learning techniques. Despite the fact that Deep Learning classifiers have dominated many research areas [70], healthcare included [71]. This could be due to the difficulties of obtaining large datasets and conducting retrospective analysis over time to train models. Furthermore, considering that interpretability is critical in healthcare [58], we can conclude that most of the studies cover the minimal requirements according to their artificial intelligence technique selection, even when they were not focused on that. SVM is the most used method—17 out of the 31 studies used SVM, and SVM has the best performance in 8 out of the 17 studies.

As regards our second research question, to identify what type of feature predictors are being used based on the literature, the list of type of data most used, from most to least used, is: clinical, patient information, molecular, and pathological images. Moreover, most of the studies combined multiple types of data, obtaining better performance to predict the risk of recurrence than when used independently. Based on clinical information, pathologic staging TNM is the most used feature composed by Node—29 out of the 31 studies—followed by Tumour and Metastasis—28 and 7 out of the 31 studies, respectively. These finding of the present study are aligned with the medical guideline [54,55]. Considering patient information, age at diagnosis is the most used feature—17 out of the 31 studies—followed by menopause status—7 out of the 31 studies. These finding are aligned with previous researches [72,73]. Based on molecular information, tumour grade is the most used feature—21 out of the 31 studies—followed by hormone receptor and tumour invasion—15 and 13 out of the 31 studies, respectively. These finding are aligned with the medical guideline [54,55]. Considering pathological images, MRI are the most used type of images—12 out of the 31 studies. These finding are aligned with the medical guideline [74]. Finally, this review confirms that consensus in the definition of feature selection and its validation over appropriate datasets is still an open problem.

In response to our third study question to identify the common training and testing methodologies, our findings cover different aspects. (i) Dataset size and class balance. Most of these studies had a limited number of patients, <1000, especially for a common disease such as breast cancer. However, number of patients developing recurrence, follow-up recurrence window, and difficulty in keeping patients records for a long time present challenges to collecting data on the risk of recurrence. None of the studies had balanced data, recurrence cases are less than 30% in all datasets [5]. Nevertheless, there are some strategies in artificial intelligence to overcome imbalanced data data augmentation [75] and synthetic data [76]. (ii) Sampling strategies. Simple random is the strategy most used in the evaluated studies—20 out of 31—which is a population selection entirely by chance. This kind of strategies could affect equity and affect the inclusion of some features during the training or testing procedures [58]. (iii) Data handling strategies. Taking into account the small datasets size for predicting the risk of recurrence, reducing size of the datasets becomes even more critical when most of the research does not deal with lack of data. This lack of data standardization also causes issues with data transfer. It makes data collection and cleansing more difficult [77]. (iv) Validation strategies. The cross-validation approach was employed in the majority of research—18 out of 31. However, because potentially biased or imbalanced data leads to a biased evaluation of a biased training model on a biased test set, this strategy cannot guarantee the quality of a Machine Learning model. An external model validation is the most recommended strategy, this was used in 11 out of 31 studies. This is directly affected due to dataset size. (v) Dataset availability. The majority of studies used private datasets—18 out of 31—that contain data from specific healthcare or research centers, affecting inclusion and generalization into the models. Additionally, this fact affects reproducibility and further comparison of the obtained results [58,61]. All these findings are aligned with the review presented by Abreu et al. [19].

Our fourth research question is to identify and critically appraise what model evaluation metrics are being used, and what are the advantages and disadvantages. Given the class imbalance present in the associated datasets, it is encouraging to find that most of the studies used specificity, sensitivity, accuracy, and AUC—20, 19, 18, and 16 out of the 31, respectively. Some studies have discussed precision and accuracy metrics in term of trustworthiness for imbalanced classes [19], as they do not effectively identify genuine positive and true negative rates. However, we found that all studies that use precision and accuracy also use complementing metrics such as specificity, sensitivity, and AUC.

Finally, none of the 31 studies shows evidence of being used in clinical practice or validated in a real-world context. Translating artificial intelligence proposals into medical practice or increasing the likelihood of them being validated in a real-world setting is still an open problem. However, artificial intelligence implementation needs to be rigorously assessed to be embraced responsibly [78,79], considering that medical errors caused by incorrect artificial intelligence are both costly and harmful. A blunder in forecasting the probability of breast cancer recurrence could have disastrous consequences for health-related quality of life and outcome [64]. However, there are some clinical practice approximations in related areas. The Food and Drug Administration (FDA) approved the cytology-based PAP smear test (Papanicolau test) using digital pathology and artificial intelligence for screening cervical cancer a while ago. However, artificial intelligence uses in routine clinical histopathology practices have been extremely limited [80]. In recent years, a new AI-based software, Paige Prostate has been developed to identify the area of prostate biopsy images with the highest likelihood of harboring cancer for further evaluation by the pathologist if the cancer is not detected on the initial review [81]. In 2021, the FDA authorized the marketing of Paige Prostate for the automated detection of cancer in prostate biopsies to assist pathologists in the detection of areas that are suspicious for cancer as an adjunct to the manual review of digitally scanned slide images. Moreover, Paige Prostate was recently tested on real-world data from a diagnostic histopathology laboratory located in a different country to classify slides into two categories: benign (no further review needed) or suspicious (additional histologic or immunohistochemical analysis required). Using Paige Prostate (in comparison to diagnosis established by two independent pathologists), the authors demonstrate that incremental improvements can be achieved in diagnostic accuracy and efficiency and that it has the potential to be employed for the automated identification of patients whose histological slides could forgo manual review by a pathologist [82]. In 2019, one of the leading clinical digital pathology service providers Phillips teamed up with Paige Prostate to bring artificial intelligence based solutions to clinical pathology diagnostics. Philips IntelliSite Pathology Solution in combination with CE marked Paige Prostate aims to provide an intuitive digital and computational pathology workflow experience to clinicians in Europe(ref-link-1). Besides the conditional approval for the US market, Philips IntelliSite Pathology Solution has market clearance in European Economic Area, United Kingdom, Ireland, and Singapore [83].

## 5. Conclusions and Future Work

Predicting the risk of recurrence in breast cancer is crucial for choosing proper treatment methods, as well as reducing morbidity and mortality [5]. Our literature search screened 492 articles to identify potentially relevant studies. The study provides an overview of artificial intelligence techniques, feature predictors, common training and testing methodologies, evaluation metrics, and systems implementation in clinical practice to predict the risk of recurrence in breast cancer. Although there are many research papers on this topic in the past decade, it remains an open problem.

On the one hand, according to this review, artificial intelligence techniques have performed well on independent and ensemble approaches, which is consistent with previous literature reviews [19]. Large datasets, on the other hand, are required to be made publicly available in order to evaluate standardised models among the various proposals. Big datasets, data augmentation, and synthetic data methodologies should be researched extensively to enable Deep Learning solutions for the prediction of risk of recurrence in breast cancer.

In summary, translation of artificial intelligence approaches into medical practice remains a challenge. However, in order to increase changes of acceptance within clinical context, artificial intelligence implementations must be thoroughly evaluated [78,79].

## Figures and Tables

**Table 1:** Exclusion and inclusion criteria applied to papers based on the purpose of our systematic review.

Exclusion	Inclusion
Papers that were not written in English	Breast cancer risk of recurrence prediction studies
Papers that were not peer-reviewed conference or journal papers (e.g., theses, dissertations, books, book chapters, pre-prints, posters, PowerPoint presentations, or other archived articles)	Studies using machine learning techniques (regression, instance-based, regularization, decision tree, Bayesian, clustering, association rule learning, artificial neural network, deep learning, dimensionality reduction, and ensemble algorithms)
Not human studies	
Surveys	

**Table 3:** Feature predictors used based on patient information. *n* corresponds to the number of studies using each feature; % is n/31×100, 31 being the total of studies included in this review.

Feature	Number (*n*)	Percentage (%)
Patient demographics		
Marital status	2	6.5
Demographic information	2	6.5
Race/ethnicity	1	3.2
Years of education	1	3.2
Personal Medical History		
Age at diagnosis	17	54.8
Menopausal status	7	22.6
Age at menarche	2	6.5
Smoking status	2	6.5
History of infertility	2	6.5
Alcohol usage	2	6.5
Death (related to breast cancer or unrelated)	2	6.5
History of other cancers	1	3.2
History of other chronic diseases	1	3.2
Breastfeeding	1	3.2
Body mass index	1	3.2
Charlson comorbidity index	1	3.2
Family history		
Breast cancer	4	12.9
Other cancers	2	6.5

**Table 4:** Feature predictors used based on clinical and molecular information. *n* corresponds to the number of studies using each feature; % is n/31×100, 31 being the total of studies included in this review.

	Feature	Number (*n*)	Percentage (%)
Anatomic staging	1. Clinical staging		
	1.1 Diagnostic imaging		
	MRI scans	12	38.7
	Ultrasonography	1	3.2
	1.2 Core biopsy	2	6.5
	2. Pathologic staging TNM		
	Nodal status	29	93.5
	Tumour	28	90.3
	Metastasis	7	22.6
	3. Post-therapy staging		
	3.1 Clinical Information		
	Radiation	10	32.3
	Hormone therapy	8	25.8
	Chemotherapy	8	25.8
	Type of surgery	7	22.6
	Therapy	4	12.9
	NAC	1	3.2
	Anti-HER2 therapy	1	3.2
	3.2 Pathological information		
	Response to neoadjuvant therapy	2	6.5
	Complete pathologic response	1	3.2
	4. Restaging in the event of tumour recurrence		
	Outcome (recurrence/not)	7	22.6
	Recurrence time	6	19.4
Prognostic stage	Tumour grade	21	67.7
	Hormone receptor	15	48.4
	Tumour invasion	13	41.9
	HER2	8	25.8
	Tumour type	7	22.6
	Ki-67	5	16.1
	Oncogene expression	2	6.5
	Multigene panels testing	1	3.2
	Other molecular markets		
	Stromal TILs	1	3.2
	CD44	1	3.2
	ABCC4	1	3.2
	ABCC11	1	3.2
	N-cadherin	1	3.2
	Pan-cadherin	1	3.2
	Cytokeratin 5/6 (CK5/6)	1	3.2
	Epidermal Growth Factor Receptor (EGFR)	1	3.2

**Table 5:** Ranking of feature predictors used based on medical images information. *n* corresponds to the number of studies using each feature; % is n/31×100, 31 being the total of studies included in this review.

Rank	Feature	Number (*n*)	Percentage (%)
3	Magnetic Resonance Imaging (MRI)	12	38.7
1	Fine Needle Aspirate (FNA)	6	19.4
4	TMA samples	1	3.2

**Table 6:** Training and testing methodologies. Excluding = excluding uncompleted data; predictive = predictive value imputation.

Publication	Publicly	Years of	Balanced	Validation	Sampling	Data Handling
	Available	Recurrence	Classes	Strategy	Strategy	Strategy
Lg et al. [22]	No	2	No	Cross validation	Simple	Expectation
						maximization
Pritom et al. [23]	Yes	—	No	Cross validation	Simple	—
Aline et al. [24]	No	5	No	Validation set	Stratified	—
Mosayebi et al. [25]	No	5	No	Cross validation	Stratified	Excluding
Alzubi et al. [26]	No	—	No	Cross validation	Stratified	Excluding
Witteveen et al. [27]	No	5	No	Validation set	Stratified	—
Cirkovic et al. [28]	No	5	No	Cross validation	Stratified	—
Ramkumar et al. [29]	No	5	No	Validation set	Stratified	Excluding
Almuhaidib et al. [30]	Yes	—	No	Cross validation	Simple	Excluding
Rosa Mendoza et al. [31]	No	2	No	—	Stratified	Excluding
Wang et al. [32]	No	5	No	70-30	Simple	Excluding
Chou et al. [33]	Yes	5	No	Validation set	Simple	Excluding
Li et al. [34]	Yes	—	No	—	Simple	Excluding
Kim et al. [35]	No	—	No	Validation set	Simple	Excluding
Kim et al. [36]	No	5	No	80-20	Simple	Excluding
Chakradeo et al. [37]	Yes	—	No	Cross validation	Simple	Excluding
Rana et al. [38]	Yes	—	No	Cross validation	Simple	Excluding
Mohebian et al. [39]	No	5	No	Cross validation	Simple	Excluding
Eun et al. [40]	No	7	No	Cross validation	Systematic	Excluding
Bhargava et al. [41]	Yes	—	No	Cross validation	Simple	Excluding
Adeyemi et al. [42]	Yes	—	No	Cross validation	Simple	Excluding
Yang et al. [43]	No	5	No	Cross validation	Simple	Excluding
Massafra et al. [44]	Yes	5–10	No	Cross validation	Simple	Predictive
Turkki et al. [45]	No	15	No	Validation set	Simple	Excluding
Kabiraj et al. [46]	Yes	—	No	Cross validation	Simple	Excluding
Sakri et al. [47]	Yes	4	No	Cross validation	Simple	Excluding
Lou et al. [48]	No	10	No	Validation set	Simple	Excluding
Ojha and Goel [49]	Yes	—	No	Cross validation	Cluster	Excluding
Kim et al. [50]	No	5	No	Validation set	Systematic	Excluding
Woojae et al. [51]	No	5	No	70-30	Stratified	Excluding
Zain et al. [52]	Yes	—	No	Cross validation	Simple	Excluding

**Table 7:** Ranking of evaluation metrics. *n* corresponds to the number of studies using each feature; % is n/31×100, 31 being the total of studies included in this review.

Rank	Feature	Number (*n*)	Percentage (%)
1	Specificity	20	64.5
2	Sensitivity	19	61.3
3	Accuracy	18	58.1
4	AUC	16	51.6
5	F-Score	8	25.8
6	Precision	7	22.6
7	Positive predictive value	4	12.9
8	Negative predictive value	4	12.9
9	Recall	4	12.9
10	Kappa statistic	2	6.5
11	Mean absolute error	1	3.2
12	Root mean squared error	1	3.2
13	Relative absolute error	1	3.2
14	Root relative squared error	1	3.2
15	Error rate	1	3.2
16	Youden’s J statistic	1	3.2
17	Standard error	1	3.2
18	Gini index	1	3.2
19	Entropy	1	3.2
20	Information gain	1	3.2

## Abbreviations

The following abbreviations are used in this manuscript:

pCR	pathological complete response
SVM	Support Vector Machines
SGDC	Stochastic Gradient Descent Classifier
WTTE- RNN	Weibull Time To Event Recurrent Neural Network
BDT	Bagged Decision Tree
LDA	Linear discriminant analysis
MRI	Magnetic Resonance Imaging
FNA	Fine Needle Aspirate
TMA	Tissue Microarray
XAI	Explainable Artificial Intelligence
FDA	Food and Drug Administration
EI	Enterprise Ireland
SFI	Science Foundation Ireland
